# Disengaging from Evil: Longitudinal Associations Between the Dark Triad, Moral Disengagement, and Antisocial Behavior in Adolescence

**DOI:** 10.1007/s10802-019-00519-4

**Published:** 2019-02-09

**Authors:** Jelle J. Sijtsema, Carlo Garofalo, Kim Jansen, Theo A. Klimstra

**Affiliations:** Department of Developmental Psychology, Tilburg University, P.O. Box 90153, 5000 LE Tilburg, The Netherlands

**Keywords:** Antisocial conduct, Moral cognitions, Dark triad, Adolescence, Development

## Abstract

Previous work has identified important correlational linkages between the dark triad of personality (Machiavellianism, psychopathy, and narcissism) and antisocial behavior in adolescence. However, little is known about the longitudinal associations between these personality characteristics and antisocial behavior, and the processes underlying these linkages. We hypothesized positive bidirectional associations between the dark triad and antisocial behavior, and that increases in moral disengagement would underlie these longitudinal associations. In the current study, we examined these hypotheses in 502 Dutch adolescents (51.8% boys, *M*age = 13.57, *SD* = 1.07) across three annual waves. Path models showed that antisocial behavior was positively associated with relative increases in moral disengagement, a general dark personality factor, and Machiavellianism specifically, but not the other way around. These paths were only observed in boys and more pronounced during the first year of the study. Finally, antisocial behavior was partly indirectly associated to psychopathy across time via antisocial behavior and moral disengagement at a later time point. Together, these findings suggest that dark personality characteristics and moral disengagement development are more likely to be the result of changes in antisocial behavior than the other way around.

**Electronic supplementary material:**

The online version of this article (10.1007/s10802-019-00519-4) contains supplementary material, which is available to authorized users.

People have a tendency to come up with reasons or excuses for their antisocial behaviors, especially when these behaviors go against their own values and norms. Such justifications are highly relevant in youth, as adolescence is characterized by a high incidence of antisocial behavior (Tremblay 2010), but also by more advanced moral reasoning (Kohlberg 1984; Lapsley and Carlo 2014). However, youth differ in the extent to which they engage in antisocial behavior and use moral justifications, as well as in the extent to which these moral justifications are associated with antisocial behaviors. Previous work has shown that youth with antagonistic personality characteristics, such as Machiavellianism, narcissism, and psychopathy, reported more antisocial behaviors compared to their peers (e.g., Klimstra, Sijtsema, Henrichs, & Cima, 2014). Although little is known about the underlying factors influencing these associations, some work has shown that these personality characteristics were also associated with poorer moral reasoning and greater use of justification of antisocial behaviors, especially in the form of moral disengagement (e.g., DeLisi et al. 2014; Shulman et al. 2011). In the current study, we thus argue that relative differences in the justification of antisocial behavior may represent an important factor explaining associations between dark triad personality characteristics and antisocial behavior. We examined this hypothesis in a sample of young adolescents.

## The Dark Triad and Antisocial Behavior

The dark side of personality is often captured with three separate, but partly overlapping characteristics referred to as the Dark Triad of personality (Paulhus and Williams 2002). These include Machiavellianism, psychopathy, and narcissism. Machiavellianism refers to the use of manipulation and strategic behavior, along with a cynical worldview and a tendency to exploit others (Baughman et al. 2012; Christie and Geis 1970). In adolescence, Machiavellian youths have also been labeled as ‘bistrategics’, because they tend to use different prosocial and antisocial strategies to get what they want, depending on the social context (Hawley 1999). Psychopathy typically includes callous and unemotional traits, low empathy, high levels of impulsivity, and a desire for sensation (Hare 1985; Paulhus and Williams 2002). Finally, narcissism can be described as the tendency to constantly crave high status and admiration, acting arrogant, and immensely enhancing the self while simultaneously devaluing others (Morf and Rhodewalt 2001; Brunell et al. 2008).

Unsurprisingly, these characteristics have been associated with antisocial and delinquent behaviors (e.g., Barry et al. 2007; Muris et al. 2017). When accounting for their overlap, recent research has shown that associations with antisocial behavior differ for the three Dark Triad characteristics (Klimstra et al. 2014; Lau and Marsee 2013). These studies showed that in adolescence, Machiavellian and psychopathic characteristics were linked to both indirect and direct antisocial behaviors, whereas narcissistic characteristics were only linked to indirect forms of antisocial behaviors (e.g., gossiping and ostracism). However, there are also some studies that reported links between narcissism and direct forms of aggression in boys (Ojanen et al. 2012), at-risk youth (Barry et al. 2018), and institutionalized youth (Muñoz Centifanti et al. 2013). There is thus some evidence for links between dark personality characteristics and different forms of antisocial behavior, but comparatively less research has investigated the longitudinal associations between these constructs and possible factors underlying these links. Identifying potential underlying factors would be important to understand developmental pathways toward antisocial trajectories and can help identify possible targets for prevention and intervention programs.

## Moral Disengagement

Cognitive processes involving moral reasoning and moral justification may act as a potentially important factor underlying the links between the dark triad and antisocial behavior. Personality is often related to how one reasons and justifies behavior, because it is linked to cognitive styles. In light of their antagonistic nature, the dark triad are likely characterized by non-normative moral cognitions. Moreover, such cognitive patterns related to moral reasoning may serve the purpose of justifying antisocial behaviors and their stable use may ultimately enhance the likelihood that such behaviors will occur again.

To this end, work focusing on moral reasoning and justifications of antisocial behavior in adolescence has typically focused on moral disengagement (Bandura et al. 1996). In his Social Cognitive Theory, Bandura (1991) explains why individuals sometimes conduct behaviors that go against their internal norms or standards and how they deal with this cognitive dissonance. According to this theory, behavior is regulated through moral norms that are learned and enforced by the social context, law enforcement, and internal values. However, when individuals engage in behavior that conflicts with these moral norms, the dissonance between what they did and how they feel about what they did, may give rise to moral emotions such as shame and guilt. It is argued that this state induces self-regulation that prevents individuals from engaging in this behavior (again). However, to avoid experiencing negative moral emotions, individuals can also circumvent the activation of self-regulation processes, by disengaging themselves from the ‘wrongness’ of their behavior. This process is referred to as moral disengagement.

To morally disengage themselves from their wrongdoings, individuals may use excuses, justifications, or rationalizations of their antisocial behavior or antisocial behaviors in general (e.g., ‘It is alright to fight when your group’s honor is threatened’, ‘It is okay to insult someone because beating him is worse’). In this way, the behavior (fighting or insulting someone) is no longer regarded as going against internal or social moral norms, and hence self-regulatory emotions, such as shame and guilt, are not experienced. Although moral disengagement strategies have been theorized to be separable along different dimensions, previous work has typically found little discriminant validity of the separate dimensions and has often looked at overall levels of moral disengagement in adolescence (Bandura et al. 1996; Obermann 2011).

Several studies have reported associations between moral disengagement and various indices of antisocial behavior in adolescence, including aggression (Hymel and Perren 2015), delinquency (Barriga et al. 2008), and externalizing behaviors in general (Bandura et al. 1996). Recent meta-analyses corroborate that youth who display more moral disengagement are more likely to be aggressive or delinquent, in both general population samples (Gini et al. 2014) and clinical forensic samples (Stams et al. 2006).

## Moral Disengagement and the Dark Triad

Some prior work has investigated empirical links between moral disengagement and the dark triad, suggesting that dark triad characteristics may be differentially associated with moral disengagement and subsequent antisocial behavior. Most work on youth’s moral disengagement has focused on psychopathy. Some researchers have argued that youth with psychopathic characteristics may be less likely to experience moral emotions such as shame and guilt (see DeLisi et al. 2014), and argued that this may be due to these youth using more moral disengagement. Moreover, it has been argued that psychopathic youth may be more prone to justifying antisocial conduct (Shulman et al. 2011). There is empirical support for these notions as several studies have shown that psychopathic traits were related to moral disengagement (O'Kane et al. 1996; Risser and Eckert 2016; Shulman et al. 2011). Moreover, Walters and DeLisi (2015) showed that psychopathic traits were indirectly linked to violence via moral disengagement. However, these authors were not able to investigate the role of relative changes in the dark triad, antisocial behavior, and moral disengagement as each construct was assessed once, at different time points.

Alternatively, it has also been suggested that psychopathic individuals need less moral disengagement, because they do not always perceive their immoral behaviors as wrong. Hence, their behavior does not elicit self-sanctions, such as guilt or shame, in the first place. Although there is no direct support for this idea, evidence from a recent meta-analysis documented only a weak relationship between psychopathic characteristics and impairments in moral reasoning in adults (Marshall et al. 2018). In interpreting these findings, the authors argued that psychopathy may only be related to subtle differences in understanding right and wrong, and hence may not predispose to more moral disengagement. In sum, it thus remains an empirical question whether there is an association between psychopathy and moral disengagement.

To our knowledge, no studies examined the links between Machiavellianism, moral disengagement, and antisocial behavior in youth. However, many of the theoretical notions pertaining to psychopathy may also hold for Machiavellianism, in light of the similarities between the two personality constructs (Miller et al. 2017). Moreover, there is some related research on adult consumer behavior supporting the idea that moral disengagement may mediate the association between Machiavellianism and antisocial behaviors (Egan et al. 2015). That is, the authors showed that psychopathy and Machiavellianism were both associated with moral disengagement, but only Machiavellianism was in turn associated with unethical consumer behavior.

Narcissism may be differently related to moral disengagement compared to psychopathy and Machiavellianism. Because of the threat of losing approval from others, antisocial behavior may be more strongly related to emotions such as shame and guilt, which increases the need for moral disengagement. To uphold a positive view to oneself and others, narcissistic youth may thus be more likely to use moral disengagement. It has also been suggested that pathological levels of narcissism may be associated to dysfunctions in morality (Kernberg 1997). Narcissists may be more likely to view others as either stupid or evil, or idolize them. In this context, narcissists may view antisocial behavior as acceptable when it is used against someone who is not worth consideration (e.g., because of being stupid or evil), or for a greater cause (e.g., loyalty to some idolized entity or group). A recent study on an adult population sample indeed found that moral disengagement was associated with a broad narcissism measure, including both grandiosity and vulnerability aspects (Fossati et al. 2014).

There are a few studies that have examined the links between narcissism, moral disengagement, and antisocial behavior, though these were limited to (young) adult samples. In a sample of undergraduates, narcissistic traits were associated with aggression and dehumanization (i.e., a type of moral disengagement with which other people’s human aspects are taken away or denied) (Locke 2009). The author also tested whether dehumanization mediated the association between narcissistic traits and aggression, but found no support for this. In another study, the link between narcissism, moral disengagement, and antisocial behavior was examined in a sample of team sport players (Jones et al. 2017). The authors found support for an indirect effect, suggesting that moral disengagement mediated the association between narcissism and antisocial behavior. In sum, there is some evidence for an association between narcissism and moral disengagement, but less support for a mediating role of moral disengagement in the association between narcissism and antisocial behavior.

## Longitudinal Associations

Despite the wealth of information on the links between the dark triad, moral disengagement, and antisocial behaviors, previous research is limited in several ways. First, little is known about the longitudinal associations between the dark triad and antisocial behavior. To date, almost all studies assessing all dark triad characteristics simultaneously examined cross-sectional data, which only allows for detecting concurrent associations, but does not provide information about how these constructs are related across time (Muris et al. 2017). The few studies that included longitudinal data only had information of each construct at one measurement occasion (e.g., personality at baseline and antisocial behavior later in time). Hence, it is unclear whether there are unidirectional or bidirectional associations between these constructs: do antisocial behaviors change the dark triad or the other way around, or is there a reciprocal association between the two? Although some scholars consider antisocial behavior to be an outcome of the dark triad (e.g., psychopathy; Cooke et al. 2004), longitudinal studies suggest that there might be a reciprocal link, such that early antisocial behavior may also predict increases in psychopathy (Forsman et al. 2010; see also: Frick et al. 2003). That is, it may be plausible that earlier engagement in antisocial behavior contributes over time to increased callousness, manipulativeness, and grandiosity. This can function either as a way of coping with the committed antisocial behavior (and related consequences such as contact with the criminal justice system or delinquent peer groups), or because of a shared underlying genetic factor of more covert (e.g., callousness) and overt (e.g., aggression) dark characteristics (see Hare and Neumann 2010).

Second, although several authors tested the mediating role of moral disengagement, all these studies were cross-sectional. They thus examined what has been termed *atemporal mediation,* leaving the order of the mediation model purely theoretical (Winer et al. 2016). However, there is much debate about the temporal order in the relationship between moral disengagement and antisocial behaviors (Maruna and Mann 2006). Whereas neutralization theories state that moral disengagement precedes the development of antisocial behavior by removing the mental barriers for antisocial conduct (Finkelhor 1984; Sykes and Matza 1957), theories about excuse making and justification suggest that antisocial behavior precedes moral disengagement (e.g., Bandura et al. 1996; Maruna and Copes 2005). Thus, longitudinal data are needed to elucidate the temporal order of the links between these constructs.

Finally, most previous work looked at one specific antisocial outcome or used a general measure of antisocial behavior, thereby obscuring potentially unique associations between the dark triad, moral disengagement, and different forms of antisocial behavior. However, there is increasing support for these unique associations, in particular regarding associations between the dark triad and antisocial behaviors (Klimstra et al. 2014; Lau and Marsee 2013). That is, whereas Machiavellianism and psychopathy seem to be associated with both direct and indirect aggression, narcissism seems to be mainly associated with indirect aggression. Moreover, all three characteristics have been associated to delinquency (Muris et al. 2017).

## The Present Study

In the current study, we aimed to address the abovementioned limitations by examining the longitudinal associations between the dark triad, moral disengagement, and antisocial behavior in adolescence. First, given previous work on the relationships between the dark triad and antisocial behavior, we hypothesized that relative changes in the dark triad were associated with relative changes in antisocial behavior over time, and vice versa. In particular, we expected that Machiavellianism, psychopathy, and narcissism were positively associated with antisocial behavior, and vice versa.

Second, based on the presence of contrasting theories (i.e., neutralization versus justification) on the temporal order of the association between moral disengagement and antisocial behavior, we hypothesized bidirectional relationships between these constructs over time.

Third, we hypothesized that the dark triad were associated with moral disengagement over time. Specifically, regarding psychopathy and Machiavellianism, theories state that links with moral disengagement may be either absent or positive. With regard to narcissism, we expected a positive association with moral disengagement, based on theory and, albeit limited, empirical evidence. Moreover, we explored whether links between the dark triad and moral disengagement were bi- or unidirectional over time.

Fourth, we hypothesized that moral disengagement would mediate associations between the dark triad and antisocial behavior. Thus, we expected that relative increases in the dark triad and antisocial behaviors would predispose to moral disengagement, and in turn would be associated with increases in the dark triad and antisocial behavior over time. However, we expected that this mediation process would be less pronounced for narcissism. Engagement in antisocial behavior in individuals with narcissistic traits might be driven by a sense of entitlement and grandiosity (e.g., being ‘above the law’), which is unrelated to moral disengagement (Fossati et al. 2014), rather than by overly immoral tendencies.

Finally, we tested for differences between boys and girls. Although boys typically report higher mean levels on all constructs, there is a lack of theoretical and empirical work on gender differences in the associations between these constructs and hence we did not have a basis to formulate specific hypotheses. However, because the relations between the dark triad and externalizing behavior tends to be consistent across gender (Klimstra et al. 2014; Sellbom et al. 2017; but see Ojanen et al. 2012), we did not expect differences between boys and girls in the examined links.

For all hypothesized associations, we ran a series of models in which we considered the dark triad characteristics separately (i.e., not accounting for shared variance), as well as a model including a general dark triad factor. The rationale for this was that, recently, concerns have been raised about partialling out shared variance between the dark triad characteristics, because the conceptual meaning of the residual variance in each characteristic may be difficult to interpret (Vize et al. 2018).

## Methods

### Participants

Data came from the longitudinal cohort Study on Personality, Adjustment, Cognition, and Emotion (SPACE). Recruitment took place at three public high schools in urban areas in the Netherlands, across various educational tracks. Public high schools in the Netherlands are accessible to youth from all socioeconomic backgrounds. Participants were followed during three annual waves. The original sample at the first wave consisted of 854 youths (49.2% boys; mean age at the first wave = 13.81, SD = 1.15), spread across the three schools with sample sizes of 169, 340, and 345 students, respectively. One school dropped out of the study, due to many students moving to a different school location. Hence, we only included participants who had information from at least two time points. Of the original sample, 502 participants (57.8%) met this requirement (51.8% boys; mean age at the first wave = 13.57 years, SD = 1.07). Compared to participants with data at only one time point, participants with data from at least two time points were significantly younger at Time 1 (Cohen’s *d =* 0.48). Results of Little’s MCAR test (Little 2012) indicated that missing data on personality, moral disengagement, and antisocial behaviors were missing completely at random (*p* = 0.15).

Of the final sample, the majority reported that their national background was Dutch (60.9%). Other national backgrounds mainly included Turkish (8.2%), Moroccan (6.5%), and Surinamese (5.7%). Moreover, at Time 1, most participants were in the first (60.0%) or second (18.6%) year of secondary school.

### Procedure

The SPACE study was approved by the local Institutional Review Board (protocol number EC-2014.41) and conducted in line with the APA ethical guidelines (American Psychological Association 2010). School principals provided permission for administering the study during school hours. Youths and their parents were informed via a detailed letter describing the content and the goals of the study. Passive consent was used to obtain parental permission, and youths provided active consent. Parents had 2 weeks to object to their child’s participation in the study. In the rare case that parents objected to their child participating in the study after administering the questionnaire, we removed all responses from that child from the study.

Questionnaires were filled out digitally or on paper during school hours under the supervision of trained psychology (under)graduate students. Participants could spend one school hour (50 min) on the questionnaire. Questionnaires were conducted in an examination setting to ensure privacy. Moreover, participants were informed that data would be processed, coded, and stored anonymously and that no one besides the researchers had access to the data.

### Instruments

#### Delinquent Behavior

Delinquent behavior was assessed at all waves with the self-reported Antisocial Behavior Questionnaire (Kretschmer et al. 2014). Participants responded on a five point scale (‘no, never’ to ‘seven or more times’) whether they had ever partaken in antisocial activities, such as stealing, fighting, and damaging things. This measure has been used in adolescent community samples in previous research, showing good internal reliability (coefficient alphas > 0.72) and significant associations to a number of individual and social risk factors (Kretschmer et al. 2014; Sijtsema et al. 2015). In the current study, exploratory factor analyses in MPlus 7.0 indicated that a two factor solution fitted the data best at Time 1 (CFI = 0.81, RMSEA = 0.056, χ^2^ = 633.537, df = 251, *p* < 0.001), yielding a delinquency factor (16 items) and a violence/ extreme theft factor (6 items), and 3 items with cross-loadings. Because violence was already assessed with the direct aggression scale (see below), we opted for the 16-item factor to represent delinquency, which showed good internal consistency between items (coefficient alphas between 0.82 and 0.88 across waves). Analyses in the Supplementary Materials further showed that this unidimensional 16-item factor yielded excellent fit at all time points and also showed measurement invariance across time and gender.

#### Aggressive Behavior

Self-reported aggression was measured at all waves with the Dutch version (Hale et al. 2008) of the Direct and indirect aggression scale (Björkqvist et al. 1992). Findings in previous studies on adolescent community samples from multiple cultures, suggested strong reliability and relevant associations of this measure with peer-reported aggression and peer victimization (Carroll and Schute 2005; Österman et al. 1998). Direct aggression was assessed with 5 items, such as ‘When I’m mad at a classmate, I will kick or strike him/her’. Indirect aggression was assessed with 12 items, such as ‘When I’m mad at a classmate, I will spread vicious rumors as revenge’. Items were scored on a 4-point scale, ranging from ‘never’ to ‘very often’. Coefficient alphas for the subscales ranged from 0.83 to 0.90 across waves.

#### Dark Triad

Youth reported on the dark triad at all waves using the Dirty Dozen (Jonason and Webster 2010). The Dirty Dozen measures narcissism (e.g., ‘I tend to seek prestige or status’), Machiavellianism (e.g., ‘I tend to manipulate others to get my way’), and psychopathy (e.g., ‘I tend to lack remorse’) with 4 items each rated on a 9-point scale ranging from 1 (‘strongly disagree’) to 9 (‘strongly agree’). Previous work showed the validity and reliability of the three factor-structure of the Dutch Dirty Dozen in its explanation of aggressive behavior in adolescents (Klimstra et al. 2014). In the current study, internal consistency was acceptable for Machiavellianism (α’s = 0.78–0.86), psychopathy (α’s = 0.68–0.81), narcissism (α’s = 0.83–0.88), and a general dark triad factor encompassing all 12 items (α’s = 0.87–0.90). Because it is not clear whether the three dark triad characteristics are truly separable or would be better represented by a single- or two-factor model (see e.g., Jonason and Luévano 2013), we also performed a range of configural tests to determine the best fitting factor-model (see Supplementary Materials). Configural tests favored a 3-factor model above 1- or 2-factor solutions at each time point and for both genders. There was one exception: a two-factor model (combining psychopathy and Machiavellianism) fitted the data best at Time 1 for boys. Specifically, the three-factor did not differ significantly in terms of model fit from this two-factor solution based on two out of three indices (∆CFI = 0.007, ∆RMSEA = 0.001; ∆χ^2^ = 6.53, *p* < 0.05). However, the three-factor model did outperform a two-factor model in tests for girls and on measurement occasions T2 and T3. Therefore, we decided to retain the three-factor model at all time points for both genders for reasons of consistency.

#### Moral Disengagement

Moral disengagement was assessed at all waves with the Moral Disengagement Questionnaire (Bandura et al. 1996). Participants rated their degree of acceptance of justification for transgressive behaviors on 32 items using a five point scale ranging from 0 (‘strongly disagree’) to 4 (‘strongly agree’). Item scores were summed and averaged with higher scores indicating more moral disengagement. Although the 32 items capture eight different types of moral disengagement with 4 items each (i.e., moral justification, euphemistic language, advantageous comparison, displacement of responsibility, diffusion of responsibility, distorting consequences, attribution of blame, dehumanization), it is often used as a unidimensional construct (see e.g., Bandura et al. 1996). Previous work provided evidence for the construct validity and reliability of this unidimensional measure in community adolescents, showing relevant associations to a number of antisocial outcomes (e.g., Bandura et al. 1996; Obermann 2011). Also in the current study, we used a unidimensional measure with good internal consistency. However, because a 1-factor solution had a poor fit to the data, we only included items with factor loadings above 0.50. This yielded a construct of 21 items with excellent internal consistency across waves (α’s between 0.91 and 0.92). At Time 1, this unidimensional configuration showed good fit: CFI = 0.91, RMSEA = 0.057, χ^2^ = 464.853, df = 181, *p* < 0.001). Moreover, this configuration showed longitudinal invariance and gender invariance at each wave (see Supplementary Materials).

### Analyses

**Fig. 1:**
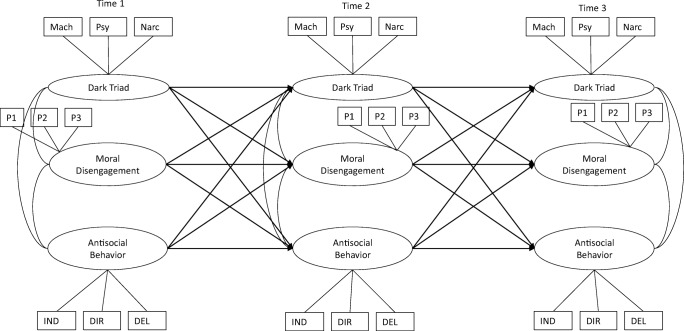
Estimated cross-lagged model

First, we calculated means and standard deviations for all study variables. To test our hypotheses we used structural equation modeling in Mplus 7.0 (Muthén and Muthén 2012). To estimate our models we used Full Information Maximum Likelihood (FIML), which accommodates missing data when missing at random, by using all available information (in contrast to list-wise deletion). To account for the skewness of our measures, we used a robust estimator (MLR). We estimated four cross-lagged panel models: one for each latent dark triad characteristic and one for the composite score of the dark triad (see Fig. 1 for an overview of the estimated model). To account for measurement error, we estimated latent factors at each time point. For Machiavellianism, psychopathy, and narcissism, we used latent factors based on the four items of each construct. Furthermore, to estimate a latent moral disengagement construct, we used three parcels of 7 items at each time point. To this end, we used the item-to-construct balance parceling method, in which factor loadings are used as a guide (Little et al. 2002). In this case, items with the highest loadings serve as anchors and are parceled with items with the lowest factor loadings. Finally, we estimated a latent antisocial behavior construct based on the observed delinquency and aggression measures, to account both for measurement error and overlap between delinquency and direct and indirect aggression.

In all models, we accounted for the within-time associations between latent constructs and the cross-time stability within latent constructs. To examine both direct and indirect associations between latent constructs across time, we included direct associations between the dark triad characteristics, moral disengagement, and antisocial behaviors, while also specifying all possible indirect paths between these constructs (e.g., from Machiavellianism at Time 1 through moral disengagement at Time 2 to antisocial behavior Time 3). With these cross-lagged panel models we examined between-person level associations between constructs. Please note that these models do not assess absolute changes, but relative changes (i.e., whether or not someone moves up in the rank-order on a variable).

Because we were also interested in whether associations of the separate dark triad characteristics could be more parsimoniously examined using a general dark triad factor, we estimated one model in which we used a latent dark triad score based on the observed scores for Machiavellianism, psychopathy, and narcissism. Finally, we reported path coefficients separately for boys and girls.

Model fit was assessed via the chi-square, the Comparative Fit Index (CFI), and the Root Mean Square Error of Approximation (RMSEA). CFIs larger than 0.90 and RMSEAs smaller than 0.08 are indicative of an adequate model fit, whereas CFIs larger than 0.95 and RMSEAs smaller than 0.05 signify good fit (Hu and Bentler 1999; Kline 2005).

## Results

Before we conducted cross-lagged path analyses, we first tested longitudinal and gender measurement invariance for our constructs (see Supplementary Materials). We established at least metric invariance for all constructs across time and gender, which is adequate for testing cross-lagged panel models (van de Schoot et al. 2012). Across time, we also established scalar invariance for all constructs, except for moral disengagement for which we only established metric invariance. This means that we could still run the cross-lagged path models, but could not interpret the mean-level changes in moral disengagement.

Moreover, we ran multi-group models by gender, comparing an unconstrained baseline multi-group model with a model in which all paths were constrained to be similar for boys and girls (see Table S6 in the Supplementary Materials). Fit indices did not significantly differ between the constrained and unconstrained models and this indicates that there are no significant differences between the coefficients for boys and girls. However, because we wanted to gain more insight into the specific coefficients for boys and girls, we presented the coefficients separately for both genders, based on the unconstrained baseline multi-group model. Furthermore, we estimated the stability within constructs over time by using earlier assessments to predict later assessments, while also accounting for the within-time associations between constructs (see Fig. 1).

### Descriptive Statistics and Correlations

Findings from Table 1 report means and standard deviations on all study variables for boys and girls, separately. In Table 2, we reported the within-time correlations for all three time points and for boys and girls separately. Correlations are based upon the cross-lagged panel models. In these models, we estimated the initial (i.e., Time 1) associations between all constructs as well as residual correlations (i.e., the within-time associations at Time 2 and 3). These residual correlations are indicative of correlated relative change (i.e., indicating that moving up in the rank order on one variable is associated with moving up in the rank order on another variable, see e.g., Neyer and Asendorpf 2001). Within-time correlations of moral disengagement with Machiavellianism, psychopathy, and the dark triad factor were positive and significant at all time points (with the exception of the correlation with boys’ Machiavellianism at Time 3). Most correlations were between 0.35 and 0.75, suggesting moderate to strong effects (Cohen 1992). Moreover, associations at Time 2 and 3 represent correlated residuals. These indicate that individuals who increased on the dark triad characteristics relative to the others in the sample, also increased in moral disengagement scores relative to the others in the sample. Correlations between moral disengagement and narcissism were lower (≤ 0.21 for boys; ≤ 0.45 for girls) compared to the other dark triad characteristics and became non-significant at later time points (at Time 2 and 3 for boys and at Time 3 for girls). This indicates that there was no correlated change. Similarly, adolescents with higher levels of Machiavellianism, psychopathy, narcissism, and dark triad factor scores reported more antisocial behavior at Time 1 and 2 (except for narcissism at Time 2). At Time 3, boys’ dark triad characteristics and girls’ narcissism were unrelated to antisocial behavior. Correlated change was thus only observed at Time 2. Finally, moral disengagement and antisocial behavior were positively correlated with each other at each time point in both boys and girls (*r*s ≥ 0.57), showing high levels of correlated relative change.

**Table 1:** Descriptive statistics and gender differences for all observed study variables

	T1 (*N* = 502)^a^				T2 (*N* = 456)				T3 (*N* = 206)			
	Boys (*n* = 253)		Girls (*n* = 241)		Boys (*n* = 228)		Girls (*n* = 227)		Boys (*n* = 106)		Girls (*n* = 100)	
	M	SD	M	SD	M	SD	M	SD	M	SD	M	SD
Machiavellianism	2.75	1.60	2.30	1.59	3.15	1.81	2.84	1.71	3.31	1.58	2.89	1.72
Psychopathy	3.03	1.57	2.80	1.65	3.44	1.74	3.22	1.78	3.76	1.79	3.00	1.83
Narcissism	3.72	1.84	3.33	1.84	4.06	1.79	3.91	1.88	3.86	1.74	3.27	1.89
Moral Disengagement	1.94	0.48	1.82	0.56	2.05	0.51	1.79	0.46	2.16	0.45	1.83	0.49
Direct aggression	1.52	0.55	1.36	0.55	1.60	0.61	1.40	0.49	1.70	0.64	1.34	0.42
Indirect aggression	1.38	0.44	1.34	0.46	1.44	0.49	1.39	0.43	1.48	0.51	1.30	0.37
Delinquency	1.53	0.54	1.46	0.60	1.64	0.68	1.43	0.44	1.51	0.64	1.31	0.41

^a^Eight participants did not report their gender

**Table 2:** Within-time (Time 1) and residual correlations (Time 2 and 3) between latent constructs

		Time 1					Time 2				Time 3					
		M	P	N	DT	MD	M	P	N	DT	MD	M	P	N	DT	MD
Boys	MD	0.39	0.38	0.21	0.43	–	0.36	0.42	*0.11*	0.38	–	*0.28*	0.38	*−0.13*	0.38	–
	ASB	0.70	0.65	0.37	0.72	0.68	0.59	0.43	0.32	0.55	0.63	*−0.06*	*0.31*	*0.21*	*0.17*	0.57
Girls	MD	0.56	0.57	0.35	0.61	–	0.52	0.55	0.45	0.65	–	0.66	0.58	*0.30*	0.75	–
	ASB	0.71	0.69	0.35	0.74	0.81	0.62	0.61	*0.33*	0.68	0.57	0.54	0.49	*0.05*	0.59	0.61

*M* Machiavellianism, *P* Psychopathy, *N* Narcissism, *DT* General dark triad factor, *MD* Moral disengagement, *ASB* Antisocial behavior. All correlations were significant at *p* < 0.05, except for those in italics. Correlations for ASB with MD were based on the model including the general dark triad factor, but were similar to the standardized correlations in the models for the separate dark triad characteristics. Residual correlation coefficients at T2 and T3 represent correlated change

### Cross-Lagged Paths

**Fig. 2:**
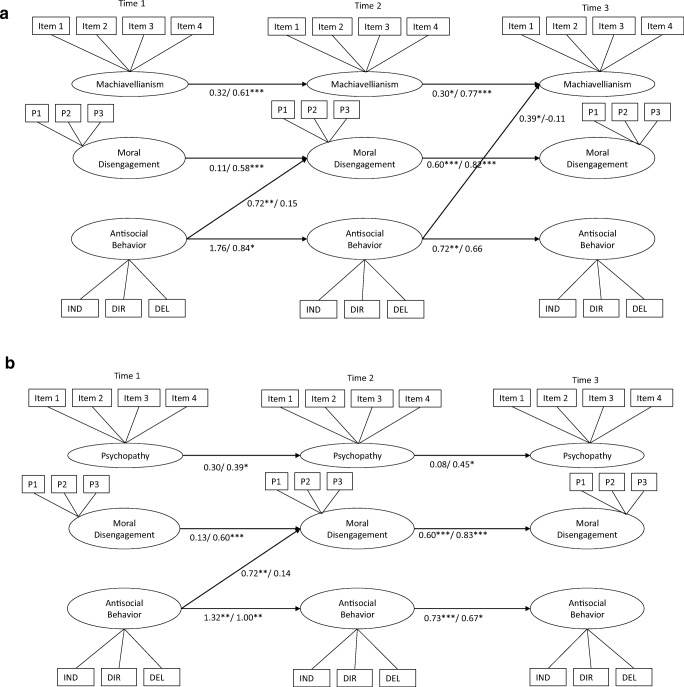

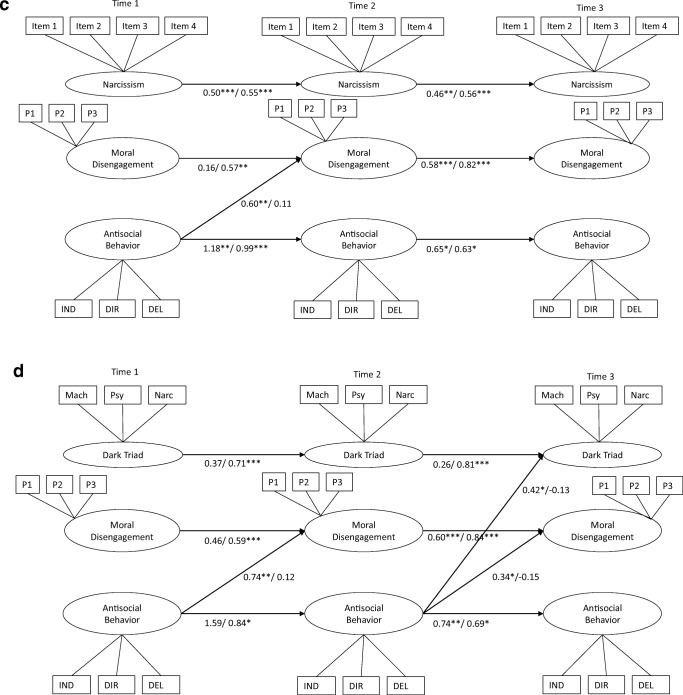
**a**–**d** Cross-lagged panel models between dark personality characteristics, moral disengagement, and antisocial behavior (within-time correlations are not displayed for ease of reading). Standardized coefficients are presented separately for boys (before slash) and girls (after slash). P1–3 = Parcel 1–3; IND = Indirect aggression; DIR = Direct aggression; DEL = Delinquency

When estimating the cross-lagged paths, we also included the stability paths of each construct across time. In most models that we estimated, there were significant levels of stability in constructs over time, though more consistently so for girls compared to boys (see Fig. 2a–d). Specifically, psychopathy, Machiavellianism, and moral disengagement did not show significant stability from Time 1 to Time 2 in boys.

#### Machiavellianism

Cross-lagged paths between Machiavellianism, moral disengagement, and antisocial behavior are presented in Fig. 2a. Fit of the unconstrained multi-group model was adequate: *X*^*2*^(773) = 1394.54, *p* < 0.001; RMSEA = 0.06; CFI = 0.90. In boys, path analysis indicated a unidirectional relationship between antisocial behavior at Time 1 and moral disengagement at Time 2. This suggests that boys who reported higher levels of antisocial behavior increased relatively in moral disengagement 1 year later, but not vice versa. Moreover, antisocial behavior at Time 2 was positively related to relative increases in Machiavellianism at Time 3, but not the other way around. In girls, there were no significant cross-lagged paths.

#### Psychopathy

Cross-lagged paths between psychopathy, moral disengagement, and antisocial behavior are presented in Fig. 2b. Fit of the unconstrained multi-group model was acceptable: *X*^*2*^(773) = 1405.43, *p* < 0.001; RMSEA = 0.06; CFI = 0.89. Similar to the findings of the model including Machiavellianism, there was a unidirectional relationship between antisocial behavior at Time 1 and moral disengagement at Time 2 in boys. There were no other significant cross-lagged paths in boys and girls.

#### Narcissism

Cross-lagged paths between narcissism, moral disengagement, and antisocial behavior are presented in Fig. 2c. Fit of the unconstrained multi-group model was acceptable: *X*^2^(773) = 1476.28, *p* < 0.001; RMSEA = 0.06; CFI = 0.89. Similar to the findings for the models including Machiavellianism and psychopathy, there was a unidirectional relationship between antisocial behavior at Time 1 and moral disengagement at Time 2 in boys. There were no other significant cross-lagged paths in boys and girls.

#### Dark Triad Factor

Cross-lagged paths between the latent Dark Triad factor, moral disengagement, and antisocial behavior are presented in Fig. 2d. The fit of the unconstrained multi-group model was acceptable: *X*^*2*^(773) = 1264.78, *p* < 0.001; RMSEA = 0.07; CFI = 0.88. In boys, path analysis indicated a unidirectional relationship between antisocial behavior and moral disengagement between both time intervals (i.e., from Time 1 to Time 2, and from Time 2 to Time 3). This suggests that boys who reported higher levels of antisocial behavior increased relatively in moral disengagement 1 year later, but not vice versa. Moreover, antisocial behavior at Time 2 was positively related to relative increases in the latent Dark Triad factor at Time 3, but not the other way around. In girls, there were no significant cross-lagged paths.

#### Indirect Effects

To examine indirect effects between the dark triad, moral disengagement, and antisocial behavior, we tested all potential indirect paths between these constructs from Time 1 to Time 3. This procedure yields a so-called *total indirect effect* that can be broken down into *specific indirect effects*, specifying via which mediator(s) the indirect pathway goes. For example, the total indirect effect between two constructs can go through both psychopathy and moral disengagement, suggesting that the total indirect consists of two specific indirect effects.

In the cross-lagged path models of Machiavellianism, psychopathy, narcissism, and the general dark triad factor, antisocial behavior at Time 1 was indirectly related to moral disengagement at Time 3 in boys, via moral disengagement and antisocial behavior at Time 2 (*b* = 0.60, SE = 0.21, *p* < 0.01). Moreover, in the models of psychopathy and the general dark triad factor, antisocial behavior at Time 1 was indirectly related to psychopathy at Time 3, via psychopathy (or the general dark triad factor), moral disengagement, and antisocial behavior at Time 2 (*b* = 0.47, SE = 0.23, *p* < 0.05). However, this indirect effect was driven largely by antisocial behavior at Time 2 (*b* = 0.34, SE = 0.25).

## Discussion

In the current study, we expanded upon previous research by assessing longitudinal associations between the dark personality characteristics, moral disengagement, and antisocial behavior to infer directionality in a sample of adolescents. This yielded several important findings. In line with our hypotheses and previous cross-sectional work on the relationships between the dark triad and antisocial behaviors (e.g., Muris et al. 2017), we found positive within-time associations between antisocial behavior and dark triad characteristics, with the exception of narcissism. These associations were moderate to strong and consistent across time and gender. Moreover, there were moderate levels of correlated changes between these constructs, suggesting that changes over time on these traits were related to each other.

In contrast to our hypotheses, there were no bidirectional associations between antisocial behavior and dark personality characteristics over time. Instead, we found unidirectional longitudinal links from antisocial behavior to the general dark personality factor and Machiavellianism. These links were more pronounced in the first year of the study and only applied to boys. This suggests that antisocial behavior predicted relative increases in the general dark personality factor and Machiavellianism, but not vice versa. This is in line with work suggesting that antisocial behaviors can be used strategically to obtain and maintain scarce resources (e.g., social status), typically seen in Machiavellian youth (Hawley 1999). Antisocial behavior may thus give access to more scarce resources and as such foster or elicit characteristics linked to keeping these resources, such as using manipulation to gain and maintain status in the peer group. Moreover, our findings are also in line with studies that showed that earlier engagement in antisocial behavior predicted psychopathic personality characteristics in youth (Forsman et al. 2010; Frick et al. 2003). Forsman et al. (2010) also found that this effect was partly genetically driven, and suggested that use of antisocial behavior could emotionally desensitize youth. Moreover, during adolescence, antisocial behaviors are often rewarded by the peer group (Dijkstra et al. 2009; Moffitt 1993) and can thus be instrumental in increasing aspects related to seeking status and attention from others (which are included in the Dirty Dozen instrument) (Jonason and Webster 2010).

In line with our hypotheses and most previous research (e.g., Egan et al. 2015; Fossati et al. 2014; Shulman et al. 2011), we showed that there are clear within-time associations between the dark personality characteristics and moral disengagement, in particular for psychopathy and Machiavellianism. This suggests that youth who report higher levels on the dark personality characteristics are more likely to exonerate their antisocial conduct, which resonates with our idea that the dark personality is characterized by non-normative moral cognitions. Regarding the associations over time, we only found correlated change, suggesting that moral disengagement and dark personality characteristics (with the exception of narcissism) change in accordance over time. However, change in moral disengagement neither predicted changes in the dark personality characteristics, nor vice versa.

Finally, we tested the direction of associations between moral disengagement and antisocial behaviors. Although we hypothesized that both directions would be plausible based upon previous research and theories, we found only support for a justification perspective and only in boys. Increases in antisocial behavior predicted relative increases in moral disengagement over time, but not the other way around. This thus suggests that boys are more likely to morally justify antisocial behavior after they have engaged in the behavior (Bandura et al. 1996) as a type of excuse making (cf. Maruna and Mann 2006). However, it should be noted that the moral disengagement questionnaire only includes so-called secondary cognitive distortions, which refer to moral justifications that are externally oriented. In contrast, researchers have also identified primary cognitive distortions that include self-serving biases, which are internally oriented (Nas et al. 2008). It could thus be reasoned that these primary cognitive distortions are more likely to *precede* antisocial behavior and thus elucidate the reverse direction.

We found no support for the hypothesis that moral disengagement mediated associations between dark personality characteristics and antisocial behaviors. This goes against moral cognitive models that would suggest that self-serving cognitions underlie the link between personality and behavior (see Dodge et al. 2006; van Leeuwen et al. 2014). If anything, cross-lagged mediation models indicated that antisocial behavior was associated with moral disengagement, which in turn was associated to psychopathy, but only in boys. This finding is more in line with a justification perspective: antisocial behavior is followed by moral disengagement (Bandura et al. 1996). This use of moral disengagement may then decrease moral emotions, such as shame and guilt, and may ultimately result in increases in psychopathy, which is marked by a lack of moral emotions. However, it should be noted that formal tests for this mediation pathway were very small and most variance of the mediation pathway went via increases in antisocial behavior at Time 2. Also, the direct longitudinal association between antisocial behavior and psychopathy was non-significant.

In sum, our findings point to strong within-time associations and correlated change between antisocial behavior, moral disengagement, and dark personality characteristics. Important to note, these associations were less pronounced for narcissism. However, we did find moderate concurrent associations between narcissism and antisocial behavior at Time 1, which is in line with previous work on cross-sectional links between narcissism and aggression (e.g., Barry et al. 2018; Muñoz Centifanti et al. 2013; Ojanen et al. 2012). Moreover, the findings support a directional effect from antisocial behavior to dark personality traits, but only in boys. These findings suggest that, at least in a general population sample of adolescent boys, psychopathy characteristics are malleable and may change as a function of both antisocial conduct and, to a lesser extent, cognitions. This aligns with other studies that focused on personality changes in general in adolescence (e.g., Borghuis et al. 2017). At the same time, the cross-lagged paths also suggest that much of the changes that occur in adolescence are unrelated to the factors discussed in the current study. This thus leaves much room for alternative explanations, for example related to contextual influences (e.g., peers, parents, life events).

The finding that cross-lagged paths from antisocial behavior to moral disengagement and dark personality characteristics was only observed in boys can have several reasons. For one, there may be more variation in these constructs in boys as compared to girls, suggesting that there is more variance to explain. This notion is strengthened when examining the stability of our constructs, which was typically higher in girls. Also generally speaking, antisocial behavior and personality traits may be better predicted in boys over time compared to girls (Moffitt 2018). Moreover, antisocial behavior may be more rewarding for boys compared to girls and hence more likely increase antisocial cognitions and personality characteristics. Previous work indeed shows that antisocial behaviors, such as aggression, are more strongly associated with high social status in boys compared to girls (Cillessen and Mayeux 2004; Cillessen et al. 2014).

A final consideration concerns the comparison of findings involving each of the dark personality characteristics separately, and findings involving a general dark personality factor. First, a similar pattern emerged in the models that included the general dark personality factor, and the Machiavellianism factor only, respectively. Thus, this may suggest that Machiavellianism is driving associations over time with antisocial behavior or that Machiavellianism is a mesh of psychopathy and narcissism, at least as measured by the Dirty Dozen. Second, the different patterns that emerged for psychopathy and narcissism, as opposed to Machiavellianism or the general dark factor, provide support for considering the three dark personality components separately, as conflating them into one super-ordinate factor may obscure such differential associations.

Our findings should be interpreted against the backdrop of some limitations. First, we solely relied on self-report measures, which may have resulted in shared-reported biases. Whereas self-reports are considered the golden standard when it comes to assessing introspective aspects such as personality and cognitions, ideally we would have used informant reports (e.g., teachers, peers) for the assessment of antisocial behaviors. Given the nature of our constructs, participants may have provided social desirable answers, by either underreporting or by boasting about antisocial aspects because they may be regarded as status enhancing. Future research may thus want to extend our findings by using informant reports of antisocial behaviors.

Second, our assessment of dark personality characteristics was limited in several ways. Using a concise personality measure such as the dirty dozen has many practical advantages (e.g., easy and quick to administer), but also results in missing out on the heterogeneity and nuances in each of the personality constructs (Miller et al. 2012). That is, narcissism and psychopathy have different facets and the Dirty Dozen may not reflect these facets to an equal extent. For example, narcissism is thought to have a grandiosity and a vulnerability dimension (e.g., Pincus and Roche 2011), but the Dirty Dozen mostly taps into the grandiosity dimension. Regarding the assessment of psychopathy, the Dirty Dozen is limited to characteristics related to callousness and lack of remorse. As such, it does not take into account more antisocial tendencies and reckless impulsivity, which are also argued to be part of psychopathy (Hare and Neumann 2010). Moreover, recently, scholars have suggested an extension of the dark personality traits by also including sadism, greed, and spitefulness (Chabrol et al. 2009; Marcus and Zeigler-Hill 2015; Paulhus 2014). Finally, there are unresolved issues of the currently used dark personality characteristics relating to measurement and construct validity, their overlap and distinctiveness, and whether these correlated characteristics truly represent a single latent construct (Glenn and Sellbom 2015; Miller et al. 2012; Muris et al. 2017). Extending the assessment of dark personality that better reflects its broadness, facets, and measurement could thus increase our understanding of antisocial developments.

Third and relatedly, means and standard deviations for moral disengagement and antisocial behaviors were relatively low, which suggest that these are low frequency behaviors or cognitions. Consequently, it is possible that some null findings are the result of little variance and/or floor effects. Replication of our study in at-risk or residential youth samples is thus warranted as associations are likely more pronounced in those samples due to more variation in both dark personality characteristics and antisocial behavior.

Fourth, it is important to note that our findings are limited to adolescence. Given the general increase in antisocial behaviors during this period (Moffitt 1993; Tremblay 2010), many of the developments observed in the current study may reflect normative changes in adolescence. As such, increases in antisocial behaviors are also, by definition, accompanied by more use of moral disengagement and increases in dark personality characteristics. Moreover, the ongoing development in adolescence of affective and cognitive empathy (Batson 2009; Hoffman 2000) may partly explain the observed developments. That is, perspective taking has been shown to increase during adolescence, and for boys only after age 15 years (van der Graaff et al. 2014). It is thus likely that both moral disengagement and psychopathic characteristics are still in development in adolescence. This also resonates with other work showing that mean levels in dark personality traits rise in adolescence (Klimstra et al. 2018). The extent to which our findings apply to childhood and adulthood thus remains a topic for future research.

In sum, we showed that longitudinal associations with moral disengagement and antisocial behaviors differed by dark personality characteristic. Awareness of these differences is thus important to consider in future research, as each characteristic may paint a different developmental picture. Whereas narcissism was largely unrelated to antisocial behavior and moral disengagement over time, Machiavellianism and psychopathy were directly or indirectly predicted by antisocial behavior in boys. Moral disengagement and antisocial behaviors were cross-sectionally associated with Machiavellianism and psychopathy in both boys and girls. Moreover, the finding that in boys antisocial behaviors precede changes in moral disengagement provides important input for the ongoing discussion about the causal link between these constructs (Maruna and Mann 2006). Although we are not able to provide hard evidence for causality, the longitudinal models suggest that moral disengagement is more likely to be a consequence of (relative) increases in antisocial behavior than the other way around.

## Electronic supplementary material

ESM 1 (DOCX 50 kb)
