# Supplementary material for: Disengaging from Evil: Longitudinal Associations Between the Dark Triad, Moral Disengagement, and Antisocial Behavior in Adolescence
Source: J Abnorm Child Psychol. 2019 Feb 9;47(8):1351–65. doi: 10.1007/s10802-019-00519-4 (PMC6617551; doi:10.1007/s10802-019-00519-4)
Supplement: Supplementary file 1 — (DOCX 50 kb) [file 10802_2019_519_MOESM1_ESM.docx]

**Supplementary material**

The types of measurement invariance that are tested should be aligned with the study goals. Therefore, we tested for configural, metric, and scalar invariance (van de Schoot et al., 2012). Configural invariance refers to the number of factors and the pattern of factor loadings being statistically equivalent across different groups or assessments. Metric invariance refers to factor loadings being statistically equivalent across groups or assessments. Finally, scalar invariance refers to intercepts of items being statistically equivalent across groups or assessments. **Invariance tests**

Because the Dark Triad was a central focus of the current study, we present detailed information about the configural invariance at each time point (S1.1) and for both genders (S1.2). That is, we compared one-, two- and three-factors models at each time point and separately for both genders. In addition, we present measurement invariance tests for the different time points (longitudinal invariance; see S2) and gender per time (gender invariance; S3 – S5) for all the measures used in the cross-lagged panel models. Because we used latent measures of Antisocial Behavior, using the Delinquency, Direct Aggression, and Indirect Aggression scales, we tested longitudinal and gender invariance for both the observed and latent constructs.

It has been recommended to rely on multiple criteria when comparing models related to measurement invariance tests (e.g., Vandenberg & Lance, 2000), because each criterion provides information on different sources of model misspecification. In the current study, we used three criteria to compare nested models: a scaled χ2-difference test (Satorra & Bentler, 2001), which should be non-significant, the delta (Δ) Comparative Fit Index (CFI), and delta (Δ) Root Means Square Error of Approximation (RMSEA), which should be <.010 and <.015, respectively (Chen, 2007). Only if at least two of these criteria were satisfied, the more restricted model should be favored over the less restricted or more complex models. For configural invariance tests, this means that we would only dismiss the one-factor model if the aforementioned criteria would favor more complex (i.e., multiple) factor models. In case of metric and scalar invariance, we would favor the more restricted models with factor loadings (for metric invariance) or item intercepts (for scalar invariance) constrained to be equal across time or gender over the models in which factor loadings or item intercepts were freely estimated for different times or groups. Absolute fit indices of the various models were also taken into account, with CFIs around .90 and larger and RMSEAs of .08 and smaller considered adequate (van de Schoot et al., 2012). However, it should be noted that these indices have a tendency to penalize models with a large number of items per latent variable (as is the case for Moral Disengagement and Delinquency) (Kenny & McCoach, 2003; Sharma, Mukherjee, Kumar, & Dillon, 2005).

**Dark Triad factor structure**

First, we established configural invariance for the Dark Triad characteristics for each time point (S1.1). At all time points, a 3-factor model fitted the data best compared to 2-factor constellations and a 1-factor model. Next, we tested configural invariance across gender at all three time points (see S1.2). At all time points, a 3-factor model fitted best for both boys and girls. However, there was one exception: at Time 1, a 2-factor model combining psychopathy and Machiavellianism items showed comparable fit indices to a 3-factor model. Because the latter factor solution showed adequate fit and for reasons of consistency, we decided to retain the 3-factor model at all time points for both boys and girls.

**Longitudinal Invariance tests**

For the Dark Triad 3-factor model, the model with factor loadings constrained to be equal over time fitted the data as well as the model without such constraints at all time points (ΔCFI < .01, ΔRMSEA < .001; see Table S2). This suggested metric invariance. When constraining intercepts to be equal across time, the model performed slightly worse, suggesting no full scalar invariance (ΔRMSEA < .01, but ΔCFI > .01, *p*-value chi-square difference test < .05; see Table S2). When items 3, 8, and 11 were estimated freely, partial scalar invariance was achieved.

For Moral Disengagement, the model with factor loadings constrained to be equal over time fitted the data as well as the model without such constraints at all time points (ΔCFI < .01, ΔRMSEA < .01, *p*-value chi-square difference test < .05; see Table S2). This suggested metric invariance. When constraining intercepts to be equal across time, the model performed slightly worse compared to the model without constraints on any of the item intercepts, suggesting that there was no scalar invariance based on two of the three criteria (ΔCFI > .010, *p*-value chi-square difference test < .05).

For Indirect Aggression, the model with factor loadings constrained to be equal over time fitted the data as well as the model without such constraints at all time points (ΔCFI = .008, ΔRMSEA = .002, *p*-value chi-square difference test >.05; see Table S2), suggesting metric invariance. When constraining intercepts to be equal across time, the model performed as well as the model without constraints on any of the item intercepts, suggesting that there was scalar invariance based on two of the three criteria (ΔCFI = .004, ΔRMSEA < .001, but *p*-value chi-square difference test < .05).

For Direct Aggression, the model with factor loadings constrained to be equal over time fitted the data as well as the model without such constraints at all time points (ΔCFI = .001, ΔRMSEA = .003, *p*-value chi-square difference test >.05; see Table S1), suggesting metric invariance. When constraining intercepts to be equal across time, the model performed as well as the model without constraints on any of the item intercepts, suggesting that there was scalar invariance based on two of the three criteria (ΔCFI = .010, ΔRMSEA = .002, but *p*-value chi-square difference test < .05).

For Delinquency, the model with factor loadings constrained to be equal over time fitted the data as well as the model without such constraints at all time points (ΔCFI < .01, ΔRMSEA < .015, *p*-value chi-square difference test <.05; see Table S2), suggesting metric invariance based on two of the three criteria. When constraining intercepts to be equal across time, the model performed as well as the model without constraints on any of the item intercepts, suggesting that there was scalar invariance based on two of the three criteria (ΔCFI = .005, ΔRMSEA < .015, but *p*-value chi-square difference test < .05).

For the latent Antisocial Behavior construct, based on Delinquency, Indirect Aggression, and Direct Aggression, the model with factor loadings constrained to be equal over time fitted the data as well as the model without such constraints at all time points (ΔCFI < .01, ΔRMSEA < .015, *p*-value chi-square difference test > .05; see Table S2), suggesting metric invariance. When constraining intercepts to be equal across time, the model performed as well as the model without constraints on any of the item intercepts, suggesting that there was scalar invariance based on two of the three criteria (ΔCFI = .008, ΔRMSEA < .015, but *p*-value chi-square difference test < .05).

**Gender invariance tests**

At Time 1, all models with factor loadings constrained to be equal for boys and girls fitted the data as well as models without such constraints (ΔCFIs ≤ .009, ΔRMSEAs ≤ .004, *p*-values chi-square difference tests >.05, except for Direct Aggression, which showed an decrease in RMSEA of .020, and Delinquency, which showed an increase in CFI of .012; see Table S3). In all cases, at least two of the three criteria for metric invariance were fulfilled.

At Time 2, all models with factor loadings constrained to be equal for boys and girls fitted the data as well as models without such constraints (ΔCFIs ≤ .004, ΔRMSEAs ≤ .010, *p*-values chi-square difference tests >.05, except for Direct Aggression, which showed a decrease in CFI of .020; see Table S4). In all cases, at least two of the three criteria were fulfilled.

At Time 3, all models with factor loadings constrained to be equal for boys and girls fitted the data as well as models without such constraints (ΔCFIs ≤ .003, ΔRMSEAs ≤ .015, *p*-values chi-square difference tests >.05, except for Direct Aggression, which showed a decrease in CFI of .039; see Table S5). In all cases, at least two of the three criteria were fulfilled.

At all three time points, models with all item intercepts constrained to be equal for men and women did not perform as well as the models without constraints on any of the item intercepts, suggesting that there was no scalar invariance. This was indicated by at least two out of three criteria for model comparisons (ΔCFIs≤.010, ΔRMSEAs≤.015, and *p*-values chi-square difference tests < .05; see Table S3 – S5). The only exception was the Dark Triad at Time 2, where the differences in CFI and RMSEA were smaller than .06 and .002, respectively.

**References**

Chen, F. F. (2007). Sensitivity of goodness of fit indexes to lack of measurement invariance. *Structural Equation Modeling, 14*, 464-504.

Kenny, D.A., & McCoach, D.B. (2003). Effect of the Number of Variables on Measures of Fit in Structural Equation Modeling. *Structural Equation Modeling, 10*, 333-351.

Satorra, A., & Bentler, P. M. (2001). A scaled difference chi-square test statistic for moment

structure analysis. *Psychometrika, 66*, 507-514.

Sharma, S., Mukherjee, S., Kumar, A., & Dillon, W. R. (2005). A simulation study to investigate the use of cutoff values for assessing model fit in covariance structure models. *Journal of Business Research*, *58*, 935-943.

Van de Schoot, R., Lugtig, P., & Hox, J. (2012). A checklist for testing measurement

invariance. *European Journal of Developmental Psychology, 9*, 486-492.

Vandenberg, R. J., & Lance, C. E. (2000). A review and synthesis of the measurement

invariance literature: Suggestions, practices, and recommendations for organizational research. *Organizational Research Methods, 3,* 4-70.

Table S1.1. *Configural Invariance Dark Triad per Time Point*

|  |  | CFI | RMSEA | χ^2^ | Df | Scaling |
| --- | --- | --- | --- | --- | --- | --- |
| Time 1 | |  |  |  |  |  |
|  | 1-factor model | .754 | .115 | 404.598 | 54 | 1.453 |
|  | 2-factor model n-m together | .772 | .111 | 377.544 | 53 | 1.413 |
|  | 2-factor model p-m together | .894 | .076 | 203.811 | 53 | 1.452 |
|  | 2-factor model p-n together | .808 | .102 | 327.093 | 53 | 1.434 |
|  | 3-factor model | .909 | .072 | 180.853 | 51 | 1.428 |
| Time 2 | |  |  |  |  |  |
|  | 1-factor model | .752 | .135 | 508.343 | 54 | 1.322 |
|  | 2-factor model n-m together | .798 | .123 | 423.052 | 53 | 1.310 |
|  | 2-factor model p-m together | .900 | .087 | 237.139 | 53 | 1.307 |
|  | 2-factor model p-n together | .789 | .126 | 439.684 | 53 | 1.272 |
|  | 3-factor model | .940 | .068 | 161.223 | 51 | 1.288 |
| Time 3 | |  |  |  |  |  |
|  | 1-factor model | .447 | .201 | 518.254 | 54 | 1.065 |
|  | 2-factor model n-m together | .720 | .144 | 288.456 | 53 | 1.265 |
|  | 2-factor model p-m together | .795 | .123 | 225.011 | 53 | 1.222 |
|  | 2-factor model p-n together | .668 | .157 | 331.767 | 53 | 1.269 |
|  | 3-factor model | .904 | .086 | 131.789 | 51 | 1.230 |

*Note*. All chi-squares were significant (*p* < .001); n = narcissism, m = Machiavellianism, p = psychopathy.

Table S1.2. *Configural Invariance across Gender*

|  |  | CFI | RMSEA | χ^2^ | Df | Scaling |
| --- | --- | --- | --- | --- | --- | --- |
| Time 1 – Boys | |  |  |  |  |  |
|  | 1-factor model | .723 | .116 | 238.257 | 54 | 1.404 |
|  | 2-factor model n-m together | .735 | .115 | 229.118 | 53 | 1.371 |
|  | 2-factor model p-m together | .862 | .083 | 145.118 | 53 | 1.396 |
|  | 2-factor model p-n together | .773 | .106 | 204.225 | 53 | 1.379 |
|  | 3-factor model | .869 | .082 | 138.511 | 51 | 1.373 |
| Time 1 – Girls | |  |  |  |  |  |
|  | 1-factor model | .765 | .121 | 243.172 | 54 | 1.369 |
|  | 2-factor model n-m together | .782 | .117 | 228.447 | 53 | 1.305 |
|  | 2-factor model p-m together | .925 | .069 | 113.515 | 53 | 1.386 |
|  | 2-factor model p-n together | .833 | .103 | 187.201 | 53 | 1.359 |
|  | 3-factor model | .944 | .060 | 95.716 | 51 | 1.354 |
| Time 2 – Boys | |  |  |  |  |  |
|  | 1-factor model | .727 | .146 | 314.989 | 54 | 1.253 |
|  | 2-factor model n-m together | .803 | .125 | 241.822 | 53 | 1.243 |
|  | 2-factor model p-m together | .901 | .089 | 148.186 | 53 | 1.243 |
|  | 2-factor model p-n together | .700 | .154 | 340.448 | 53 | 1.047 |
|  | 3-factor model | .963 | .055 | 86.155 | 51 | 1.231 |
| Time 2 – Girls | |  |  |  |  |  |
|  | 1-factor model | .752 | .135 | 278.813 | 54 | 1.324 |
|  | 2-factor model n-m together | .780 | .129 | 252.799 | 53 | 1.307 |
|  | 2-factor model p-m together | .882 | .094 | 160.441 | 53 | 1.301 |
|  | 2-factor model p-n together | .817 | .117 | 218.739 | 53 | 1.328 |
|  | 3-factor model | .915 | .082 | 128.295 | 51 | 1.281 |
| Time 3 – Boys | |  |  |  |  |  |
|  | 1-factor model | .318 | .194 | 267.265 | 54 | 1.030 |
|  | 2-factor model n-m together | .710 | .128 | 143.785 | 53 | 1.277 |
|  | 2-factor model p-m together | .735 | .122 | 135.770 | 53 | 1.234 |
|  | 2-factor model p-n together | .571 | .155 | 187.341 | 53 | 1.256 |
|  | 3-factor model | .856 | .092 | 96.149 | 51 | 1.229 |
| Time 3 – Girls | |  |  |  |  |  |
|  | 1-factor model | .580 | .208 | 287.565 | 54 | 1.071 |
|  | 2-factor model n-m together | .682 | .183 | 229.897 | 53 | 1.090 |
|  | 2-factor model p-m together | .865 | .119 | 128.061 | 53 | 1.099 |
|  | 2-factor model p-n together | .744 | .164 | 195.322 | 53 | 1.162 |
|  | 3-factor model | .927 | .089 | 91.547 | 51 | 1.109 |

*Note*. All chi-square tests were significant (*p* <. 01).

Table S2. *Longitudinal Invariance Tests*

|  |  | CFI | RMSEA | χ^2^ | Df | Scaling |
| --- | --- | --- | --- | --- | --- | --- |
| Dark Triad (3 factor model) | |  |  |  |  |  |
|  | Baseline model | .918 | .041 | 979.492 | 534 | 1.134 |
|  | Metric Invariance | .913 | .041 | 1024.948 | 552 | 1.135 |
|  | Scalar Invariance | .894 | .045 | 1151.689 | 576 | 1.129 |
|  | Partial Scalar Invariance  (item 3, 8 and 11 free) | .903 | .043 | 1098.689 | 570 | 1.131 |
| Moral Disengagement | |  |  |  |  |  |
|  | Baseline model | .855 | .039 | 3157.220 | 1809 | 1.0940 |
|  | Metric invariance | .854 | .038 | 3204.156 | 1849 | 1.0932 |
|  | Scalar invariance | .830 | .041 | 3472.447 | 1889 | 1.0920 |
| Indirect Aggression | |  |  |  |  |  |
|  | Baseline model | .889 | .043 | 887.213 | 456 | 1.3779 |
|  | Metric invariance | .897 | .041 | 877.091 | 476 | 1.4209 |
|  | Scalar invariance | .893 | .041 | 911.168 | 496 | 1.4037 |
| Direct Aggression | |  |  |  |  |  |
|  | Baseline model | .935 | .053 | 264.898 | 111 | 1.3616 |
|  | Metric invariance | .936 | .050 | 272.164 | 121 | 1.4264 |
|  | Scalar invariance | .926 | .052 | 306.405 | 131 | 1.3966 |
| Delinquency | |  |  |  |  |  |
|  | Baseline model | .821 | .043 | 1933.293 | 1014 | 1.3464 |
|  | Metric invariance | .829 | .041 | 1922.254 | 1044 | 1.3977 |
|  | Scalar invariance | .824 | .041 | 1980.490 | 1073 | 1.3876 |
| Total Antisocial Behavior | |  |  |  |  |  |
|  | Baseline model | .979 | .045 | 30.559 | 15 | 1.8741 |
|  | Metric invariance | .988 | .031 | 28.428 | 19 | 2.0945 |
|  | Scalar invariance | .980 | .037 | 37.213 | 22 | 1.9770 |

*Note*. All chi-square tests were significant (*p* < .001); Although several CFI fit indices are slightly below conventional cutoffs (.90) it has to be noted that these indices have a tendency to penalize models with a large number of items per latent variable (Kenny & McCoach, 2003; Sharma, Mukherjee, Kumar, & Dillon, 2005).

Table S3. *Gender Invariance Tests* *at Time 1*

|  |  | CFI | RMSEA | χ^2^ | Df | Scaling |
| --- | --- | --- | --- | --- | --- | --- |
| Dark Triad ^a^ | |  |  |  |  |  |
|  | Baseline multigroup | .901 | .074 | 251.134 | 106 | 1.370 |
|  | Metric Invariance | .901 | .072 | 261.403 | 115 | 1.357 |
|  | Scalar Invariance | .890 | .072 | 288.528 | 127 | 1.327 |
| Moral Disengagement | |  |  |  |  |  |
|  | Baseline multigroup | .917 | .055 | 634.376 | 362 | 1.2058 |
|  | Metric invariance | .919 | .054 | 649.996 | 382 | 1.1973 |
|  | Scalar invariance | .900 | .058 | 731.080 | 403 | 1.1870 |
| Indirect Aggression | |  |  |  |  |  |
|  | Baseline multigroup | .955 | .056 | 152.045 | 86 | 1.6988 |
|  | Metric invariance | .955 | .053 | 162.622 | 96 | 1.7007 |
|  | Scalar invariance | .944 | .056 | 188.342 | 106 | 1.6353 |
| Direct Aggression | |  |  |  |  |  |
|  | Baseline multigroup | .959 | .091 | 48.598 | 16 | 1.6482 |
|  | Metric invariance | .968 | .071 | 47.099 | 21 | 1.8228 |
|  | Scalar invariance | .955 | .076 | 62.440 | 26 | 1.6972 |
| Delinquency | |  |  |  |  |  |
|  | Baseline multigroup | .923 | .045 | 297.809 | 200 | 1.9424 |
|  | Metric invariance | .935 | .040 | 296.690 | 215 | 1.9826 |
|  | Scalar invariance | .907 | .046 | 349.841 | 232 | 1.9316 |

*Note*. All chi-squares were significant (*p* < .001). ^a^ Residual correlation between items 9 and 10 in all models.

Table S4. *Gender Invariance Tests* *at Time 2*

|  |  | CFI | RMSEA | χ^2^ | df | Scaling |
| --- | --- | --- | --- | --- | --- | --- |
| Dark Triad (3 factor model) ^a^ | |  |  |  |  |  |
|  | Baseline multigroup | .908 | .086 | 273.388 | 102 | 1.255 |
|  | Metric Invariance | .911 | .081 | 277.173 | 111 | 1.253 |
|  | Scalar Invariance | .906 | .079 | 298.386 | 123 | 1.233 |
| Moral Disengagement | |  |  |  |  |  |
|  | Configural invariance | .863 | .067 | 723.308 | 362 | 1.2166 |
|  | Metric invariance | .864 | .065 | 740.385 | 382 | 1.2148 |
|  | Scalar invariance | .831 | .070 | 825.289 | 405 | 1.2045 |
| Indirect Aggression | |  |  |  |  |  |
|  | Configural invariance | .846 | .097 | 266.834 | 86 | 1.6279 |
|  | Metric invariance | .842 | .093 | 282.083 | 96 | 1.7151 |
|  | Scalar invariance | .811 | .097 | 328.046 | 106 | 1.6379 |
| Direct Aggression | |  |  |  |  |  |
|  | Configural invariance | .972 | .071 | 33.916 | 16 | 1.7205 |
|  | Metric invariance | .952 | .081 | 51.574 | 21 | 1.8102 |
|  | Scalar invariance | .930 | .088 | 70.964 | 26 | 1.6710 |
| Delinquency | |  |  |  |  |  |
|  | Configural invariance | .830 | .066 | 394.026 | 200 | 1.7032 |
|  | Metric invariance | .849 | .060 | 387.144 | 215 | 1.7962 |
|  | Scalar invariance | .788 | .069 | 474.894 | 233 | 1.7663 |

*Note*. All chi-squares were significant (*p* < .001). ^a^ Residual correlation between items 9 and 10, 9 and 11, and 5 and 7 in all models.

Table S5. *Gender Invariance Tests* *at Time 3*

|  |  | CFI | RMSEA | χ^2^ | df | Scaling |
| --- | --- | --- | --- | --- | --- | --- |
| Dark Triad (3 factor model) ^a^ | |  |  |  |  |  |
|  | Baseline multigroup | .887 | .098 | 194.651 | 98 | 1.123 |
|  | Metric Invariance | .895 | .090 | 196.612 | 107 | 1.132 |
|  | Scalar Invariance | .875 | .094 | 225.648 | 119 | 1.125 |
| Moral Disengagement | |  |  |  |  |  |
|  | Configural invariance | .810 | .081 | 604.260 | 362 | 1.1257 |
|  | Metric invariance | .814 | .078 | 619.444 | 382 | 1.1255 |
|  | Scalar invariance | .775 | .083 | 692.012 | 405 | 1.1210 |
| Indirect Aggression | |  |  |  |  |  |
|  | Configural invariance | .791 | .110 | 192.840 | 86 | 1.4688 |
|  | Metric invariance | .825 | .095 | 185.344 | 96 | 1.6497 |
|  | Scalar invariance | .793 | .099 | 211.952 | 106 | 1.5911 |
| Direct Aggression | |  |  |  |  |  |
|  | Configural invariance | .723 | .112 | 457.910 | 200 | 1.4580 |
|  | Metric invariance | .684 | .116 | 509.809 | 215 | 1.5094 |
|  | Scalar invariance | .663 | .115 | 547.643 | 233 | 1.5390 |
| Delinquency | |  |  |  |  |  |
|  | Configural invariance | .880 | .150 | 53.263 | 16 | 1.2730 |
|  | Metric invariance | .813 | .164 | 79.222 | 21 | 1.3174 |
|  | Scalar invariance | .730 | .177 | 109.812 | 26 | 1.2248 |

*Note*. All chi-squares were significant (*p* < .001). ^a^ Residual correlation between items 1and 2, 9 and 10, 9 and 11, 5 and 6, and 5 and 7 in all models.

Tabel S6. *Model comparisons between constrained and unconstrained models*

|  |  | CFI | RMSEA | *χ*^2^ | *df* | Scaling |
| --- | --- | --- | --- | --- | --- | --- |
| Baseline model | |  |  |  |  |  |
|  | Latent Dark Triad | .918 | .053 | 716.742 | 295 | 1.1543 |
|  | Machiavellianism | .936 | .043 | 723.092 | 376 | 1.1924 |
|  | Psychopathy | .922 | .046 | 775.982 | 376 | 1.1558 |
|  | Narcissism | .918 | .050 | 851.185 | 376 | 1.1455 |
| Baseline multigroup | |  |  |  |  |  |
|  | Latent Dark Triad | .882 | .066 | 1264.779 | 608 | 1.0477 |
|  | Machiavellianism | .897 | .057 | 1394.541 | 773 | 1.0661 |
|  | Psychopathy | .886 | .058 | 1405.425 | 773 | 1.0591 |
|  | Narcissism | .888 | .061 | 1476.282 | 773 | 1.0473 |
| Constrained model | |  |  |  |  |  |
|  | Latent Dark Triad | .873 | .066 | 1370.977 | 664 | 1.0833 |
|  | Machiavellianism | .881 | .059 | 1556.870 | 835 | 1.0949 |
|  | Psychopathy | .875 | .058 | 1526.864 | 835 | 1.0848 |
|  | Narcissism | .866 | .064 | 1671.280 | 835 | 1.0743 |

*Note*. All chi-square tests are significant at *p* < .001.
